# Vestibulocochlear Neuritis as a Paradoxical Reaction in an Immunocompetent Patient with Tuberculous Meningitis

**DOI:** 10.3390/diagnostics15243179

**Published:** 2025-12-12

**Authors:** Sekai Tsujimoto, Koji Hayashi, Mamiko Sato, Yuka Nakaya, Toyoaki Miura, Yasutaka Kobayashi

**Affiliations:** 1 Department of Rehabilitation Medicine, Fukui General Hospital, 55-16-1 Egami-cho, Fukui 910-8561, Japan; 2 Graduate School of Health Science, Fukui Health Science University, 55-13-1 Egami, Fukui 910-3190, Japan

**Keywords:** tuberculosis, meningeal, vestibulocochlear nerve diseases, meningitis, cranial nerves

## Abstract

A 30-year-old previously healthy man presented with fever and headache. HIV tests yielded negative results. Cerebrospinal fluid (CSF) analysis revealed pleocytosis (619/µL), elevated protein (210.3 mg/dL) and adenosine deaminase levels, and decreased glucose levels. A positive CSF culture for tuberculosis confirmed the patient had tuberculous meningitis (TBM). He was treated with methylprednisolone, isoniazid, rifampicin, pyrazinamide, and ethambutol (all highly sensitive). His compliance with medication was good. After six weeks of treatment, he was discharged in stable condition. Eight weeks after onset, he was readmitted with vertigo and right deafness. CSF examination showed worsened pleocytosis (819/µL) and protein levels (4296.1 mg/dL). Contrast-enhanced MRI revealed enhancement of meninges in the brainstem and spinal cord as well as the right vestibulocochlear nerve. No brain abscesses were observed. Based on these findings, a paradoxical reaction (PR) with vestibulocochlear neuritis following antituberculous therapy initiation was suspected. He received oral prednisolone, leading to rapid resolution of vestibulocochlear symptoms within two days. Although cranial nerve enhancement due to PR has been mentioned in the literature, specific imaging demonstrating it is scarce. This case highlights PR as a cause of cranial neuropathy in TBM and provides clear radiological evidence of direct inflammatory spread to the vestibulocochlear nerve, bridging a gap in the current literature.

**Figure 1:**
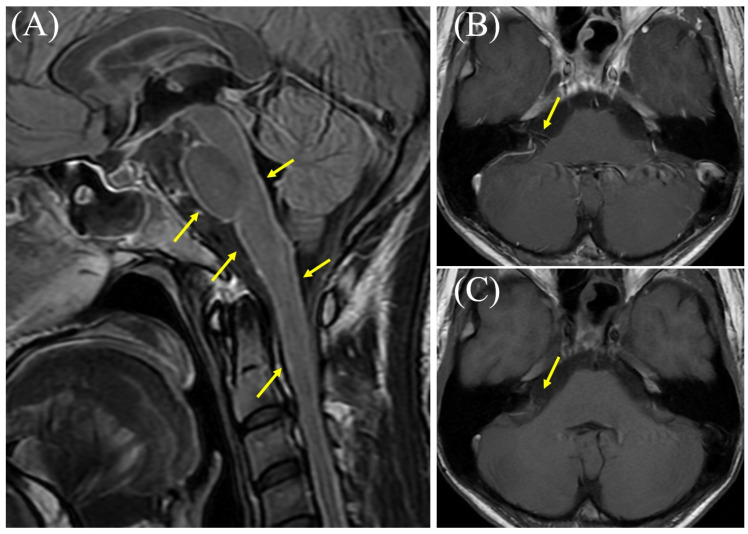
Brain magnetic resonance imaging (MRI) results obtained eight weeks after onset. (**A**) Contrast-enhanced T2-fluid attenuated inversion recovery MRI showing hyperintense signals in the brainstem meninges (arrows). (**B**) Contrast-enhanced T1-weighted MRI showing enhancement of the right vestibulocochlear nerve (arrow). (**C**) Non-contrast MRI of the same region (arrow). Cranial nerve (CN) involvement is a common and serious complication of tuberculous meningitis (TBM), the most severe form of tuberculosis (TB) [1,2]. The incidence of this complication has been reported to range from 25% to 50%, with a large retrospective study from China documenting cranial nerve palsy in 33.3% of 114 TBM patients [1,2]. One major study identified the oculomotor nerve (CN III) as the most frequently affected area (52.2% of palsy cases), followed by the optic nerve (CN II) (28.3%) and the abducens nerve (CN VI) (10.9%) [1]. Palsy of the vestibulocochlear nerve (CN VIII) appears to be relatively rare, affecting approximately 4.3% of TBM patients [1]. Etiologies of CN involvement related to TBM are diverse, including extensive exudative basal arachnoiditis, vascular infarction, basal cistern inflammation with obstructive hydrocephalus, tubercular abscesses (tuberculomas), and adverse effects of antituberculosis therapy (ATT) (e.g., streptomycin; CN VIII or ethambutol; and CN II) [3,4]. In our case, the patient was treated with appropriate antituberculosis medications (including isoniazid, rifampicin, pyrazinamide, and ethambutol) as well as methylprednisolone. Eight weeks after onset, he developed vertigo and right-sided deafness, accompanied by worsened cerebrospinal fluid findings. Detailed otolaryngological assessment during readmission revealed right hearing loss, right tinnitus, aural fullness in the right ear, and rotatory vertigo. Examination revealed left-beating nystagmus, present with and without visual fixation, which was visually suppressed. Audiometry confirmed right sensorineural hearing loss (SNHL); the four-frequency pure tone average (PTA4) was 53.8 dB in the right ear and 20.0 dB in the left ear upon readmission. Objective quantitative vestibular function tests, such as the head impulse test (HIT), video head impulse test (VHIT), or calorics, were not performed. Additionally, contrast-enhanced brain MRI showed enhancement of the meninges and cranial nerve VIII. Although the examination was suboptimal due to patient movement, this cranial nerve enhancement was not observed during the first admission (Figure S1). Types of CN involvement mentioned in previous studies were ruled out, including extensive basal exudative arachnoiditis, inflammatory vasculopathy with infarction, basal cisternitis with obstructive hydrocephalus, tuberculomas, and ATT-related adverse effects. Based on these findings, the spread of inflammation to the right vestibulocochlear nerve (vestibulocochlear neuritis) due to a paradoxical reaction (PR) following the initiation of ATT was suspected. He received oral prednisolone, which led to a rapid resolution of vestibulocochlear symptoms within two days, and an improvement in the severity of SNHL was observed seven days after the initial measurement, with the right PTA4 increasing significantly to 20.0 dB (left PTA4: 18.3 dB). PR is a complication of TB treatment where a patient’s condition worsens despite receiving appropriate ATT [5,6,7]. This phenomenon is defined by the clinical or radiological worsening of pre-existing TB lesions or the development of new ones in patients who initially showed improvement following treatment [5,6,7]. It is considered a diagnosis of exclusion, meaning other causes for deterioration like treatment failure, drug resistance, poor compliance, or a secondary infection must be ruled out [5,6]. PR has been reported in HIV-negative individuals and is thought to result from immune system restoration, as TB infection is controlled by ATT [5,7]. This phenomenon typically occurs within several months (usually less than 4 months) of treatment initiation [6,8,9]. The exact mechanism of PR is not fully understood, but it is believed to be immunologically mediated [10,11]. The leading theories suggest that it results from an exaggerated immune response to TB antigens [6,12]. As for treatment, the most critical first step in managing PR is to continue the prescribed ATT regimen [6,13]. PR is an inflammatory response to mycobacterial antigens, not a sign of treatment failure, drug resistance, or relapse [13]. Therefore, altering or discontinuing an ATT regimen is generally not recommended and may be hazardous [8,13]. In non-severe PR cases, symptomatic treatment and observation are recommended [9]. In contrast, in severe PR cases, immunosuppressants including steroids are used and often lead to rapid clinical and radiological improvement [9,10]. Although a few reports have mentioned cranial nerve enhancement associated with PR, few reports provide corresponding images related to PR. A prospective cohort study of TBM patients in Indonesia found that, among 33 patients who experienced radiological worsening—likely due to PR—24% (8 patients) developed new cranial nerve enhancement [8]. In this study, an example of oculomotor nerve enhancement was shown in baseline imaging, but images of cranial nerve enhancement related to PR were not provided. In another study, MRI findings associated with PR included nodular leptomeningeal enhancement in the basilar cisterns and a right sylvian fissure, in addition to a fissure along cranial nerves; however, specific images were not provided [5]. Our case, therefore, significantly contributes to the existing knowledge by providing clear neuroradiological evidence of direct inflammatory spread to the vestibulocochlear nerve during a PR in TBM. This unique pictorial evidence visually confirms the clinical manifestation of vestibulocochlear neuritis as PR, thereby bridging a critical gap in the imaging literature and enhancing our understanding of this challenging complication. There is a limitation in this report. While the diagnosis of vestibulocochlear neuritis was supported by clinical findings (vertigo, nystagmus) and MRI evidence of CN VIII enhancement, objective quantitative assessments of vestibular function, such as the head impulse test, VHIT, or caloric testing, were not conducted. This omission represents a limitation with respect to fully characterizing the extent of the peripheral inner-ear involvement.

## Data Availability

The data presented in this study are available on request from the corresponding author. Due to patient privacy and ethical considerations, the data are not publicly accessible.

## Supplementary Materials

The following supporting information can be downloaded at: https://www.mdpi.com/article/10.3390/diagnostics15243179/s1, Figure S1: Brain MRI findings obtained at first admission.

## Abbreviations

The following abbreviations are used in this manuscript:

MRI	Magnetic resonance imaging
TB	Tuberculosis
TBM	Tuberculous meningitis
CN	Cranial nerve
ATT	Antituberculous therapy
PR	Paradoxical reaction
ART	Antiretroviral therapy
