# Supplementary material for: Vestibulocochlear Neuritis as a Paradoxical Reaction in an Immunocompetent Patient with Tuberculous Meningitis
Source: Diagnostics (Basel). 2025 Dec 12;15(24):3179. doi: 10.3390/diagnostics15243179 (PMC12731431; doi:10.3390/diagnostics15243179)
Supplement: Supplementary file 1 [file diagnostics-15-03179-s001.zip › diagnostics-3929206-supplementary.pdf]

diagnostics

MDPI

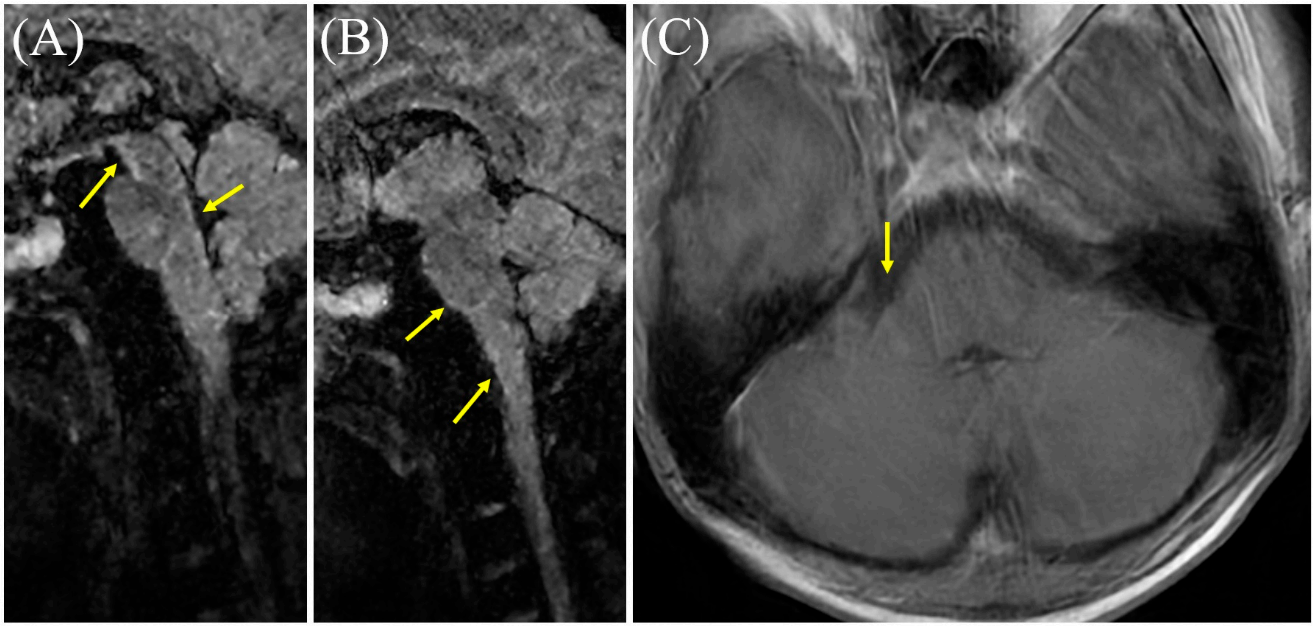

Supplementary Figure S1: Brain MRI findings on the first admission. The patient was in pain and very mobile, resulting in a suboptimal MRI study. Meningeal enhancement is observed (arrows) (A, B), while no enhancement of the eighth cranial nerve was detected (arrow) (C) within the observable range.
